# Biology before the SOS Response—DNA Damage Mechanisms at Chromosome Fragile Sites

**DOI:** 10.3390/cells10092275

**Published:** 2021-09-01

**Authors:** Devon M. Fitzgerald, Susan M. Rosenberg

**Affiliations:** Departments of Molecular and Human Genetics, Biochemistry and Molecular Biology, Molecular Virology and Microbiology, and Dan L Duncan Comprehensive Cancer Center, Baylor College of Medicine, Houston, TX 77030, USA

**Keywords:** chromosome fragile sites, DNA damage, DNA repair, double-strand break repair, Holliday junctions, topoisomerase, DNA structures

## Abstract

The *Escherichia coli* SOS response to DNA damage, discovered and conceptualized by Evelyn Witkin and Miroslav Radman, is the prototypic DNA-damage stress response that upregulates proteins of DNA protection and repair, a radical idea when formulated in the late 1960s and early 1970s. SOS-like responses are now described across the tree of life, and similar mechanisms of DNA-damage tolerance and repair underlie the genome instability that drives human cancer and aging. The DNA damage that precedes damage responses constitutes upstream threats to genome integrity and arises mostly from endogenous biology. Radman’s vision and work on SOS, mismatch repair, and their regulation of genome and species evolution, were extrapolated directly from bacteria to humans, at a conceptual level, by Radman, then many others. We follow his lead in exploring bacterial molecular genomic mechanisms to illuminate universal biology, including in human disease, and focus here on some events upstream of SOS: the origins of DNA damage, specifically at chromosome fragile sites, and the engineered proteins that allow us to identify mechanisms. Two fragility mechanisms dominate: one at replication barriers and another associated with the decatenation of sister chromosomes following replication. DNA structures in *E. coli*, additionally, suggest new interpretations of pathways in cancer evolution, and that Holliday junctions may be universal molecular markers of chromosome fragility.

## 1. Bacterial to Human Biology, per Miroslav Radman: A Personal Introduction

Miroslav Radman is a force of nature, personality and imagination, which is why one of us (SMR, narrator of this introduction) accepted his invitation to his lab post-PhD and prior to a previously arranged postdoc. My PhD mentor, Frank Stahl, said about the decision to go there that one of his colleagues had described Miro as “a DNA polymerase without a proofreading subunit.” Mutations, however, allow evolution. So, that among other aspects of Miro’s style was why, Paris notwithstanding, my mere six months with Miro at Institut Jacques Monod, Université Paris VII was like rain on a garden, and continues to influence my lab’s output.

There was a lot going on (in Miro’s head), including the birth of his idea that homologous recombination (HR) between slightly diverged “homeologous” DNA sequences would be prevented by mismatch repair, which would destroy the heteroduplex-DNA HR intermediate by binding base mismatches and killing those intermediates (unwinding or other). That is, that mismatch repair edits and reduces recombination “errors” that cause genome rearrangement, as he [1,2], Matthew Meselson and colleagues [3], as well as Paul Modrich [4] and others, had shown that it did for DNA replication. Miro thought that the ability to recombine DNA was a reasonable molecular definition of species, similar to the ability to produce reproduction-competent offspring as a functional definition of sexual species. His lab showed this in a series of exciting papers beginning with *Escherichia coli* interspecies recombination with Salmonella, which they showed was possible in mismatch repair-deficient mutants [5]. Leroy Worth, Paul Modrich and he also demonstrated biochemically that MutS and MutL mismatch-repair proteins inhibit RecA recombinase activity in model HR reactions between diverged (homeologous) DNA sequences [6]. Later, bringing his other baby, the SOS response [7], to bear on the recombinational genetic barrier between species, his then PhD student Ivan Matic and he demonstrated that SOS helps dismantle the barrier between species, mostly by its upregulation of RecA recombinase [8]. The two opposing forces regulate genome [9,10] and species evolution, they proposed, by controlling both the extent of sequence divergence, which mismatch repair reduces and SOS mutagenesis promotes [11], and of recombinational genetic isolation [5], promoted by mismatch repair [5] and reduced by SOS [8].

Some impressions of Miro’s long-ago lab at Jussieu:

(1) Not only could Miro try lots of wild ideas, but others did too. Miro’s lab was like permission to play in a raucous, exciting game—with Miro!
(2) If he had dozens of bad ideas and one good one per day, overall, that was a win. Eradicating bad ideas is easier, and, arguably, can be taught. Some ideas that prove useful were just gifts, and it was ok to give them to oneself and others, who would tolerate the clunkers.
(3) The media kitchen would not make my media, nor let me into their domain to make them myself, because American postdocs are inconsequential in the French system of tenured technicians. So, six months produced only a modest hole in my CV.

Miro also impressed me with his lab’s and collaborators’ ability to move their discoveries, conceptually and experimentally, from phage lambda and *E. coli* directly into mouse [12,13], and to early human genome analysis [14]. Although molecular biologists use(d) simple genetic models such as bacteria, phages, and yeast to illuminate the basics of life, most were not working in bacteria and human simultaneously. A notable exception is Richard Kolodner and colleagues’ demonstration that humans have homologs of bacterial MutS and MutL mismatch repair proteins and their loss of function is mutagenic and cancer-driving [15].

The review that follows covers the mechanisms behind genome-destabilizing and human disease-instigating chromosome fragile sites, as inferred from the identification of fragile sites and mechanisms in *E. coli* [16]. The impression Miro made is likely to have influenced my lab’s: previous discovery of proteins of the human “DNA damageome” via an *E. coli* screen and identification of homologous proteins in humans that act similarly in human cells and cancers [17]; demonstration and use of a new role of *E. coli* RecQ DNA helicase to predict mechanistic features of human cancer evolution [18], also discussed here; efforts to create a universal toolbox of engineered proteins that capture DNA reaction intermediates in bacterial and human living cells, e.g., [18,19]; and work, over the whole of the lab’s operation, on stress-induced mutagenesis as a model for cancer and evolution across the tree of life [20,21,22], including bacterial antibiotic resistance [23]. The idea that one might move bacterial discovery to humans, without waiting for others, was at least partly planted by my immensely enjoyable scientific association with Miro. His current work on parabiosis [24], and human aging proteomes, and how to slow the process by inference from radiation- and desiccation-resistant bacteria and tardigrades [25], continues his style of freedom from dogmata, directly extrapolating from microbe to human, never being boring, and radiating the excitement of finding fundamental new biology.

## 2. DNA-Damage and -Repair Intermediates in Bacteria Illuminate Cancer

Much of mutagenesis is preceded by DNA damage [17,22], making the origins of DNA damage one of the farthest upstream events in evolution of genomes, cancers, and organisms. Spontaneous DNA-damage mechanisms may, therefore, illuminate how, when and where evolution begins, may be regulated [17,22], and potentially may be inhibited therapeutically, (e.g., [21,22,23,26,27,28]). In any chemistry, the reaction intermediates define the mechanism.

Spontaneous DNA damage and repair intermediates can be elusive in living cells because of their rarity and transience. To capture transient reaction intermediates, we engineer proteins that bind, trap, and label specific DNA structures (e.g., [16,17,18]), either as fluorescent foci or for enrichment by chromatin immunoprecipitation and sequencing (ChIP-seq) or similar methods [29,30].

### 2.1. Trapping Four-Way DNA Junctions

**Figure 1:**
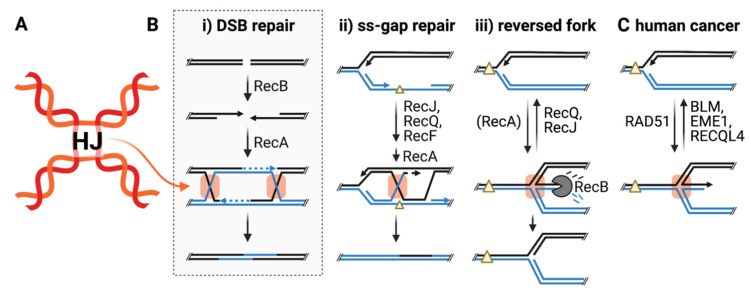
Holliday junction structure and formation. (**A**) An X-shaped Holliday junction (HJ). (**B**) Three pathways of HJ formation, highlighting key components of each pathway. HJs are highlighted by orange boxes. Close parallel lines, basepaired DNA strands; yellow triangles, replication-blocking DNA damage. Gray box indicates HJ-forming pathway at fragile sites. (**i**,**ii**) [16]. (DSB repair reviewed [33]). (**iii**) Replication fork stalling and reversal in *E. coli.* (RecA) indicates that although reversed forks occur without RecA [31,32], RecA overproduction induces higher levels of fork reversal [18]. (**C**) Proposed roles [18] of RAD51 in promoting fork reversal or stabilizing reversed forks, and BLM, EME1, and RECQL4 preventing or removing reversed forks. These are suggested, first, by RAD51 orthology to RecA, which promotes reversed forks [18]. Second, the many common cancers that overexpress *RAD51* also show strongly correlated upregulation of RNAs of both HJ-resolution proteins (EME1, GEN1) and BLM and RECQL4 homologs of RecQ helicase, which prevents fork reversal, presumably allowing DNA replication [18].

We used a catalytically dead mutant of *E. coli* RuvC 4-way DNA junction-specific endonuclease fused to GFP (RuvCDefGFP, or RDG) to bind and trap 4-way DNA junctions, also known as Holliday junctions (HJs) (Figure 1A), with high specificity [18]. HJs are transient X-shaped DNA structures with four duplex arms (Figure 1A). HJs form during the homology-directed repair (HDR) of DNA double-stranded breaks (DSBs, Figure 1Bi) and single-stranded (ss) gaps (Figure 1Bii), and when stalled replication forks are remodeled (reversed, Figure 1Biii) [31,32]. We quantified spontaneous RDG (HJ) foci in living cells, and found that most spontaneous HJ foci arise during repair of ssDNA gaps; the focus appearance depends on the ssDNA-gap repair proteins [18], shown in Figure 1Bii. The spontaneous HJ foci also appear dependently on DNA replication, which suggests that the spontaneous ssDNA gaps result from replication.

### 2.2. Proteins That Promote or Reduce Stalled-Fork Structures

We also learned that overproduction of the universal recombinase RecA provokes HJ-foci caused by replication-fork stalling and reversal, and that RecQ DNA helicase, the founding member of the conserved RecQ helicase family, prevents replication-fork reversal (Figure 1Biii) [18]. The promotion of reversed forks via RecA was observed in biochemical experiments with purified proteins and model DNA substrates [34], but not in living cells. In cells, reversed forks occur independently of RecA [31,32], but are nevertheless increased by excess RecA [18]. Extra-abundant RecA might impede forks by excessive binding to ssDNA at the fork, leading to fork stalling and reversal [34].

### 2.3. Bacterial Proteins Suggest Reversed-Fork RNA Signature in Cancer Transcriptomes

The unexpected finding that RecQ DNA helicase prevents, and RecA promotes, fork reversal in *E. coli* [18], prompted us to look in cancer transcriptomes at the mRNAs of the orthologous proteins. RecQ has five human homologs; it was assumed that the loss of function of each is genome-destabilizing and cancer promoting [35] due to a reduction of HDR leading to genome instability (mutations and genome rearrangements). Human RAD51 is a RecA ortholog and is upregulated in many diverse cancers [36].

What if human RAD51 also promoted replication-fork stalling and reversal in cells? How could the many RAD51-overproducing cancers proliferate and replicate their DNA with stalled reversed forks?

**Figure 2:**
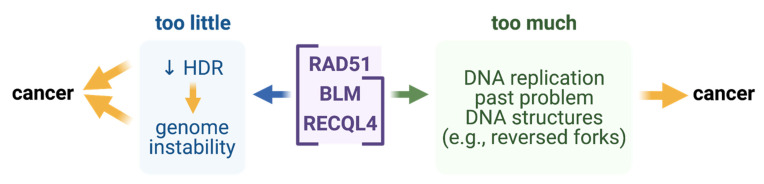
Hypothesis: Proteins that promote or prevent reversed replication forks needed at “Goldilocks” levels for cancer avoidance. Findings on—(1) the promotion of reversed forks in cells by *E. coli* RecA (RAD51 ortholog) overproduction, and their prevention by RecQ [18]; and (2) the strong correlations in cancers of RecQ-orthologous BLM and RECQL4 mRNA levels with RAD51 mRNAs [18]—suggest that “too much” BLM or RECQL4 proteins might allow DNA replication by averting replication-fork reversal, promoted by RAD51 overproduction, which is seen in many cancers [18]. “Too little” RAD51, BLM, or RECQL4 is cancer-driving due to various mechanisms that instigate genome instability, which drives cancer evolution [35].

We looked for suggestions in human cancer transcriptomes of mRNAs of human proteins that might remove or prevent reversed forks, and thus allow *RAD51*-overexpressing cancers to replicate [18]. We found strong correlations of increased human RAD51 mRNA levels with increased mRNA levels of two components of Holliday-junction resolution pathways, EME1 and GEN1, suggesting that four-way junctions are indeed increased and these resolvases might remove them, and so allow DNA replication. We also found strong correlations of mRNAs of RecQ homologs, BLM and RECQL4 with RAD51 mRNA [18]. This suggests the possibility that overexpression of these RecQ homologs might promote cancers, and might do so by reducing levels of reversed forks caused by increased RAD51, thus allowing DNA replication [18] (hypothesis in Figs 1C and 2). The strong association of RAD51 mRNA with BLM mRNA was seen in eight of the eight most common cancers: breast, lung, acute myeloid leukemia, colon, kidney, thyroid, bladder, and prostate [18]. EME1, RECQL4, and GEN1 mRNAs were correlated strongly with high RAD51 in six, four, and two of the eight most common cancers, respectively [18]. These proteins might be required by cells at “Goldilocks” levels: too little driving cancer by genome instability, and too much allowing cancers to replicate DNA and proliferate (Figure 2).

### 2.4. Seeing Holliday-Junction Genomic Landscapes with X-seq

We mapped HJs in the genomes of *E. coli* strains using ChIP-seq of RDG, called X-seq, which we tested in cells with enzymatically induced site-specific DSBs [18]. The HJs that accompany DSB repair flanked the DSB site at Chi (recombination hotspot, [33]) sequences [18]. Chi sites reduce the RecBCD exonuclease activity in favor of loading RecA onto DNA, leading to HJs and repair nearby [37].

## 3. Spontaneous “Fragile Sites” in the *E. coli* Genome

Where in bacterial genomes do spontaneous, endogenous DNA damage and repair occur? Are there hotspots akin to eukaryotic chromosomal fragile sites?

### 3.1. HJs at E. coli Genomic Fragile Sites

**Figure 3:**
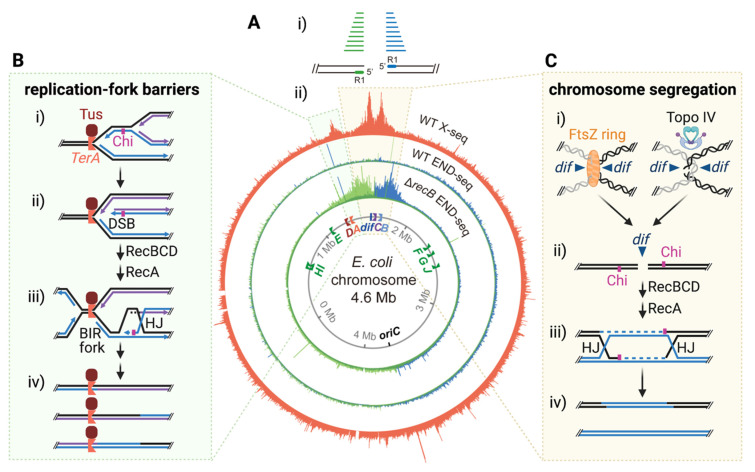
Genome-wide mapping of spontaneous HJs and DSBs reveal three major *E. coli* fragile sites. (**A**) (**i**) Diagram of END-seq color coding, green and blue indicate DSB-end polarity. (**ii**) Circular maps of spontaneous HJs (orange), obtained via X-seq [16,18], and DSB ends located using END-seq [38], in DSB repair-proficient wild-type (WT) and repair-deficient ∆*recB* cells [16]. DSBs at *Ter* sites are one-ended; DSBs surrounding *dif*, the site of chromosome decatenation, appear to be two-ended. (One-ended DSBs in different cells in the population are also possible.) (**B**) Tus-*Ter* replication-fork barriers cause fork collapse and one-ended DSB formation (**i**,**ii**) [16], a “stop-and-wait” model. (**iii**) DSBs are repaired by replication primed by DNA-break repair (break-induced replication or BIR) and converging forks (**iv**). (**C**) *dif*-proximal DSBs form during cell division, probably by guillotining by the FtsZ ring (septum) and/or topoisomerase (Topo) IV half-reactions (**i**) [16]. DSBs are repaired by HR in daughter cells after cell division occurs (**ii**–**iv**). Dashed lines, newly synthesized DNA.

We performed X-seq in proliferating *E. coli* and identified three spontaneous X-seq peaks, which represent sites of spontaneous recurrent HJs (Figure 3A, orange track) [16]. The three HJ hotspots occur in the region of replication termination (Figure 3Aii, orange track). All three arise dependently on the proteins required for initiation of homology-directed repair (HDR) of DSBs (Figure 1Bi), implicating their origins in DSB repair. Appearance of the spontaneous HJ peaks required RecA recombinase, specifically its HR function, and the DSB-end-specific RecBCD nuclease, which “resects” DSB ends and loads RecA, beginning HDR (Figure 1Bi). The spontaneous HJ hotspot peaks do not require RecF [16] (Figure 1Bii) and so are unlikely to result from repair of ssDNA gaps. The RecBCD-dependence of the HJ peaks rules out the possibility that they might be reversed replication forks (Figure 1Biii) as follows.

Despite the occurrence of some of the HJ peaks at replication barriers—*E. coli Ter* sites—the recurrent spontaneous HJ hotpots cannot be reversed replication forks because their appearance depends on RecBCD nuclease [16] (Figure 1Bi), which removes and destroys reversed forks (Figure 1Biii) [31,32]. This destruction is so potent that before the development of the HJ-trapping proteins [18], which bind and protect HJs from degradation, all previous reports of reversed forks in *E. coli* were made in RecBCD nuclease-deficient mutant cells; it was impossible to detect fork reversal in RecBCD-proficient cells (see [31,32] and many subsequent papers). The RecBCD-dependence excludes fork reversal and supports the origin of the HJ peaks as intermediates in DSB repair (Figure 1Bi). Their origin in DSB repair is also supported by the HJs’ locations at clusters of Chi DSB repair-promoting sites [16].

### 3.2. DSB Ends near the HJs

A molecular definition of fragile sites could be sites of recurrent DNA damage and/or repair. We adapted human-cell DNA-end sequencing, END-seq [38], for DSB mapping in *E. coli*, and, with it, found that the recurrent HJs are associated with recurrent DSB ends (Figure 3Ai,Aii, blue and green inner tracks) [16]. We consider these sites to be bacterial fragile sites. These fragile sites are located in the replication terminus region and display two distinct mechanisms of fragility (Figure 3B,C).

### 3.3. Fragility at Replication Barriers

Replication barriers are created by Tus protein binding to the *TerA* site (Figure 3Aii, green highlight, and B), which displays a major HJ peak and a one-ended DSB peak (Figure 3A). *TerA* is one of 10 sites in the *E. coli* genome that are bound tightly by Tus to form unidirectional barriers to replication forks. These sites are oriented such that replication forks that cross the terminus region are stopped, which prevents replication of the circular chromosome “backwards” towards the origin. Presumably, the *Ter* sites stall early-arriving forks until a slower fork can arrive from the other direction to terminate replication (Figure 3Biii,Biv).

We found that appearance of recurrent DSBs and HJs near *TerA* requires the Tus barrier protein and DNA replication from the chromosomal origin (*ori*) [16]. In addition, the TerA-proximal DSBs are one-ended, implicating replication fork collapse (Figure 3Bii) [16]. We propose a “stop-and-wait” model in which a one-ended DSB forms when one replication fork stalls at *TerA* and a subsequent replication fork, from the next round of replication, arrives before the first stalled fork is resolved (Figure 3Bi,Bii). In the growth conditions used, multiple rounds of replication occur between cell divisions, making this type of event likely.

It is possible that the one-ended DSBs at *TerA* form through other mechanisms, such as cleavage of the stalled fork by a nuclease or reversal of the stalled fork, followed by cleavage by RuvABC. However, these possibilities are unlikely, partly because there are no known *E. coli* nucleases with fork- (three-way junction) cleaving activity. Moreover, reversed replication forks are detectable by X-seq [18] and would be increased in the absence of RecB, because RecBCD degrades reversed forks (Figure 1Biii). The TerA-proximal X-seq signal, however, is eliminated instead of amplified in ∆*recB* cells, again supporting their origin as intermediates in DSB-end repair (Figure 1Bi,Biii).

After a DSB end forms, RecBCD degrades it to the nearest cluster of Chi sites and then loads RecA to promote strand exchange, HJs, which we detected, and break-induced replication (BIR) [39] (Figure 3Biii). The BIR fork will either generate another one-ended DSB when it reaches *TerA* and initiate the next cycle of futile repair, or can be resolved by two replication forks coming from the opposite direction (Figure 3Biii,Biv).

Interestingly, we detected one-ended DSBs at some other *Ter* sites, but did not detect recurrent HJs nearby [16]. This might reflect the more even distribution of Chi sites near the other *Ter* sites, resulting in more diffuse HJs that are not visible as peaks above genome-wide background. Similar cycles of DSB formation and failed repair might be occurring at other *Ter* sites too.

Although Tus-*Ter* barriers are thought to have evolved to stabilize the genome by preventing replication “backwards” in the chromosome halves, they can also destabilize the genome by promoting recurrent DNA damage and repair, i.e., become fragile sites. Perhaps this is a matter of growth conditions, with Tus-*Ter* barriers stabilizing genomes in “natural” slow growth conditions and destabilizing genomes only during rapid laboratory growth conditions. The *Ter* region was observed by Jean-Michel Louarn and colleagues to be a massive HR hot zone [40], which we expect was due to these fragile sites.

This mechanism might, at first glance, appear to be specific to bacterial chromosomes with *Ter* sites and Tus protein, but it is not. Human genomes also carry replication barriers and “late replicating DNA.” For example, barriers occur at regions of unusually structured DNA such as at ribosomal RNA genes, and these are sites of detectable DNA breakage [41]. Similarities between human fragile sites and this mechanism are outlined in Section 4.

### 3.4. Fragility at the Site of Chromosome Decatenation

The other fragile site consists of HJs and DSB ends that flank the *dif* site (Figure 3Aii, yellow highlight), where chromosome decatenation (Figure 3Ci) and dimer resolution typically occur. Both processes separate sister chromosomes that are either catenated (interlinked) after replication, or covalently linked by a crossover, to allow segregation into daughter cells. Linear sister chromosomes of eukaryotes must also be decatenated for segregation; thus, this mechanism is likely to apply to humans as well, discussed in Section 4.

Unlike DSBs at *Ter* sites, the DSB ends surrounding *dif* were detectable only in repair-deficient ∆*recB* cells (Figure 3Aii), implying that they are repaired efficiently in wild-type cells [16]. The DSB ends detected in ∆*recB* cells are consistent with formation of two-ended DSBs at or very near *dif* (Figure 3Cii), followed by erosion of DSB ends by nucleases (other than RecBCD in ∆*recB* cells). The *dif*-proximal DSB ends and HJs are abolished when cells are treated with cephalexin, an inhibitor of cell division, which is needed for chromosome segregation to occur [42,43]. We propose a model in which spontaneous failure of decatenation and/or dimer resolution results in DSB formation at *dif* [16] (Figure 3C). Decatenation is performed by type-II topoisomerase (Topo) IV [44]. Type-II topos break both DNA strands then re-ligate them. Given their high frequencies, the DSBs probably form from Topo IV half reactions (DNA breakage without ligation) on catenated sister chromosomes (Figure 3C). Less frequently, XerCD site-specific recombinase might generate the DSBs. XerCD resolves covalent dimers to monomers beginning with cleavage of both DNA strands during cell division (Figure 3Ci) [43]. The HJ signal broadens and increases moderately in strains that lack *dif* or components of the XerCD resolvase, probably because of increased chromosome tearing in cells with reduced resolution capability [16]. The *dif*-proximal DSBs are repaired by HR to form HJs at the nearest clusters of Chi sites on either side of *dif* (Figure 3Ciii) [16]. DSB repair presumably occurs in daughter cells, which contain more than one chromosome copy at cell division during rapid growth.

The three fragile sites are estimated to break and generate HJs in 1–3% of genome replications [16]. These estimates are higher than previously reported for chromosomes that are “guillotined” by the cell-division machinery at *dif* (~0.3% [45]), probably because of the increased sensitivity of our assays.

Our estimates for fragile-site breakage are similar to our previous estimates of overall DSB frequency in growing *E. coli* [19,46], suggesting that most spontaneous DSBs in *E. coli* are localized to the terminus region. Other models are possible. Overall, proteins such as RDG [18] and DSB-detecting phage Mu Gam [19], and the END-seq method [38] allow sensitive, precise and direct detection of transient DNA damage and repair intermediates that were previously undetectable in cells.

### 3.5. Previous Observations of Terminus Pathology

Previous studies indicated that the terminus region is prone to over-replication or loss of double-stranded DNA—both potentially genome destabilizing—in the absence of certain proteins. DNA repair factors such as RecG helicase [47], SbcCD hairpin nuclease and ExoI nuclease [47,48], and RecBCD [47,49] appear to facilitate the accurate completion of replication by processing DNA intermediates formed during the convergence of replication forks, and thus prevent genomic instability in the region. Additionally, cells that lack RecBCD suffer dramatic loss of double-stranded DNA in the terminus region, proposed to result from the accumulation of σ-replicating chromosomes [50,51]. We note two important differences between these previous findings and the ones highlighted above. First, previous studies have relied on less direct and/or lower resolution measurements of DNA damage such as cell viability, DNA marker frequency by next-generation sequencing, and labeling of genomic loci with fluorescent proteins. Second, previous studies focused on terminus instability in mutant cells, whereas our findings [16] suggest that substantial spontaneous DNA damage and repair occur in the terminus region normally in wild-type cells, with specific DNA intermediates identified. Without further study, it is unclear whether these observations can be reconciled into a single model of terminus maintenance, or whether they reflect processes that are occur only in specific mutant strains, growth conditions, etc.

### 3.6. Possible Genome Instability

Does the constant churn of DNA damage and repair in the terminus region leave behind heritable traces? This is the deal maker or breaker for the analogy to human fragile sites, which are associated with disease mutations [52,53]. Data from bacterial mutation accumulation (in repair-defective mutants) and population-sequencing studies suggest that the terminus region might be preferentially susceptible to base substitutions and transposable-element insertions (discussed, [16]). A definitive demonstration of possible terminus-specific mutability, its relationship with mutagenic DNA break repair [20,21,22], and potential impact on *E. coli* genome evolution, remain to be determined.

## 4. Human Fragility Mechanisms and the Bacterial Models

Aspects of the mechanisms of chromosome fragility discussed above may appear to be specialized to bacteria, but a closer look suggests that they could be universal. Human cells display multiple types of chromosomal fragile sites, with the most well-studied being common fragile sites (CFSs) [52], which are frequently associated with neurological disease- and cancer-driving genome alterations [52,53].

### 4.1. “Stop-and-Wait” Model at Replication Barriers

Although the specific bacterial Tus-*Ter* replication barrier is not present in human cells, the fragility modeled when a fork pauses (e.g., Figure 3B) is likely to occur at other difficult-to-replicate DNA regions, common to bacterial and eukaryotic genomes. Eukaryotic ribosomal RNA loci contain site-specific replication fork barriers involving bound proteins, unusual DNA structures, or both [54], and are associated with DSB formation [41]. Moreover, many CFSs contain AT dinucleotide repeats and/or long poly(dA:dT) tracts, which drive DSB formation, probably by replication-fork stalling and collapse [41,55,56]. Additionally, both CFSs [57] and bacterial fragile sites [16] are associated with under-replicated DNA; the forks paused at Tus-*Ter* are “waiting” for the neighboring replichore to be completed, and the CFS regions may result, at least in part, from site-specific replication fork stalling. Furthermore, DSB formation and DSB repair occur at the boundaries of under-replicated fragile site DNA [53,57], as we show they do in *E. coli* (Figure 3A and [16]). Finally, the seemingly least “eukaryotic” aspect of the “stop-and-wait” model in Figure 3B is the proposed second round of replication, which creates a DSB end (Figure 3Bi,Bii). Although apparently improbable in eukaryotic S phase, many oncogenes dysregulate the cell cycle and allow for over-replication in cancer cells [58], where this could occur.

### 4.2. Dangerous Decatenation Model

The fragility caused by apparent failures of chromosome segregation and/or decatenation (Figure 3C), seems probable in human chromosomes. All chromosomes become interwound during replication, and must be decatenated using similar type-II topoisomerases [59]. That is, chromosome catenation is a problem of semi-conservative replication of B-form DNA, not a peculiarity of circular chromosomes. If half reactions of a type-II topoisomerase cause the DNA breaks, this problem would be expected to be general to double-stranded DNA genomes, including in linear chromosomes. A hint that humans might have a similar mechanism(s) is suggested by the recent finding that human topoisomerase, TOP3A, is implicated in the promotion of DSB formation and the resolution of chromosome bridges at CFSs [60]. Moreover, a topoisomerase-interacting protein, TOPBP1, is associated with improved stability at fragile sites [61,62], suggesting to us that topoisomerase half reactions might underly the fragility of CFS. TOPBP1 binds and might modulate the activity or fidelity of the TOP2A type II-topoisomerase, which decatenates human sister chromosomes. Additionally, type II topoisomerase half-reactions are a source of spontaneous DSBs at many sites in eukaryotic genomes [63], and the *E. coli* terminus region may provide a good model for further investigation of topoisomerase-mediated DNA damage.

## 5. Concluding Thoughts

Our use of X-seq to identify spontaneous fragile sites in the *E. coli* chromosome suggests that HJs could be a universal molecular marker for chromosome fragility [16]. HJ-detection reagents such as RuvCDefGFP and techniques such as X-seq are far more sensitive than cytological methods, and the use of “trap” proteins such as RuvCDefGFP could cause transient events to leave a permanent marker. This might additionally allow the discovery and examination of spontaneous fragility in human cells, in contrast with the current reliance on replication-stressor drugs to increase the frequency of fragility. A similar protein to RuvCDef was used to demonstrate HJ structures in telomere T-loops [64]. Work is ongoing to develop and optimize similar broadly useful HJ-trapping reagents that can be applied generally, including in human cells.

Genome-wide detection of recurrent HJs has the potential to reveal sites of replication-fork stalling and reversal, recurrent HDR, and genome-destabilizing repair pathways such as microhomology-mediated break-induced replication (MMBIR) [65], which, in *E. coli*, requires HJ-specific processing proteins [66].

The link between chromosome fragility and genome instability at human CFSs, and possibly in the *E. coli* terminal genomic DNA, hints at how endogenous biology shapes the mutation landscape of cells. In all of the organisms investigated, local mutation rates vary ~10-fold on the scale of kilobases to megabases [67,68]. Many mechanisms contribute to local variation in mutation rates, including base-specific enzymatic activity (e.g., APOBEC-mediated cytidine deamination in ssDNA [69]), chromatin features [68], and nuclear architecture [70]. However, the known mechanisms, in combination, are not sufficient to explain the total mutation rate variation observed in the human genome [67]. Many as-yet-unknown processes are likely to contribute to local variations in mutation rate, which, we suggest, will be found to occur via localized hotspots of DNA damage and repair [16], and possibly as a result of stress-inducible mutagenic break repair [22]. Model organisms such as *E. coli*, and the superb tools that are possible with it, will continue to reveal fundamental mechanisms of genome (in)stability and genome evolution.

## Data Availability

Not applicable.
